# Sex-Specific Reversal of Stress-Induced Depressogenic Behaviors by Inhibition of HMGB1, TLR4, and NF-κB Signaling

**DOI:** 10.1007/s12035-026-05908-7

**Published:** 2026-05-06

**Authors:** Aslıhan Bahadır-Varol, Bengisu Solgun, Gülce Küreli, Emre Cem Esen, Emine Eren-Koçak

**Affiliations:** 1 Institute of Neurological Sciences and Psychiatry, Hacettepe University, Sihhiye, Ankara, 06100 Turkey; 2 Department of Psychiatry, Faculty of Medicine, Hacettepe University, Ankara, Turkey; 3 Faculty of Medicine, Izmir School of Economics, İzmir, Turkey

**Keywords:** Acute stress, Sex differences, HMGB1, NF-κB, Anterior cingulate cortex, Nucleus accumbens

## Abstract

Acute stress and inflammatory mediators are linked to stress-related mental disorders, yet sex-specific differences in the molecular pathways connecting them remain poorly understood. Male and female C57BL/6 mice underwent predator stress while restrained. Apathy/anhedonia and behavioral despair were assessed using the splash test (ST) and tail suspension test (TST). Neuroinflammatory signaling was assessed by immunohistochemistry through measurements of HMGB1 release and NF-κB nuclear translocation in the anterior cingulate cortex (ACC) and the nucleus accumbens (NAc). To further assess the roles of HMGB1, NF-κB and TLR4 in acute stress–induced behavioral changes in both sexes, mice were administered the respective inhibitors prior to stress, and changes in depression-like behaviors were subsequently assessed. Finally, microglial numbers and cleaved caspase-1 levels were evaluated by immunohistochemistry, and microglial ramifications were assessed by Sholl analysis. Acute stress induced increased immobility in the TST and decreased grooming in the ST in both sexes. Although stress triggered comparable increases in HMGB1 release and NF-κB translocation in both sexes, pharmacological inhibition of HMGB1, TLR4, and NF-κB exerted antidepressant effects exclusively in males. In females, none of the inhibitors was effective in reversing acute stress-induced immobility in TST and decreased grooming in ST. Although the numbers of microglia recruited by acute stress were similar in both sexes, they were more ramified in females regardless of stress exposure. Cleaved caspase-1 levels were elevated only in males after stress exposure, without any alteration in females. These findings underscore sex-specific differences in acute stress–induced alterations in the brain. The results suggest that downstream signaling pathways may differ by sex or that females possess protective mechanisms that constrain the behavioral consequences of neuroinflammation. This study highlights the necessity of considering sex as a critical biological variable in developing interventions for stress-related disorders.

**Supplementary Information:**

The online version contains supplementary material available at 10.1007/s12035-026-05908-7.

## Introduction

Stress is a major environmental risk factor for the development of stress-related psychiatric disorders such as depression and anxiety [1]. Sex is an important variable in stress responses and vulnerability to affective disorders. Women experience higher rates of stress-related disorders, such as depression and anxiety, while men display distinct symptomatology and neurobiological alterations [2, 3]. Activation of neuroinflammatory signaling pathways is one of the key mechanisms through which stress contributes to the development of psychiatric disorders [4, 5]. Neuroinflammatory signaling pathways show sex-specific divergence in response to stress, with associated differences in behavioral and molecular outcomes [6, 7]. Nevertheless, most animal studies investigating the effects of stress have been conducted exclusively in males, which limits the generalizability of the findings to females and consequently, their translational relevance to women.

HMGB1-TLR4–NF-κB signaling has been implicated in the neuroinflammatory mechanisms underlying stress-induced behavioral and affective disturbances [8]. HMGB1 acts as a DNA chaperone, a chromatin stabilizer, an autophagy inducer, and an anti-apoptotic factor. When released, it acts as a damage-associated molecular pattern (DAMP) (i.e., alarmin), initiating immune signaling through Toll-like receptor 4 (TLR4) and activating the NF-κB pathway [9–11]. Nuclear factor kappa-B (NF-κB) is a proinflammatory transcription factor bound to the inhibitor of NF-κB (I-κB) that keeps it inactive in the cytoplasm [12]. Inflammatory signals, like binding of HMGB1 to TLR4, activate I-κB kinase (IKK), which phosphorylates I-κB leading to its proteasome-mediated degradation and translocation of NF-κB into the nucleus.

Despite growing evidence linking neuroinflammation to stress-related pathologies, potential sex differences in these molecular pathways remain poorly understood. In the present study, we investigated the effects of acute stress on depression-like behavior and examined the possible involvement of HMGB1 and NF-κB signaling in stress-induced behavioral alterations in the anterior cingulate cortex (ACC) and nucleus accumbens (NAc) in male and female mice. The ACC and NAc are key nodes in neural circuits underlying stress-related psychiatric disorders, particularly those governing behavioral despair and reward processing [13–15].Consistent with the functional roles of these regions, depression-like behaviors were assessed using the ST and TST, paradigms widely employed to model self-grooming and behavioral despair. In parallel, activation of neuroinflammatory signaling was examined via HMGB1 release and nuclear NF-κB translocation within the ACC and NAc, enabling the investigation of molecular alterations in brain regions directly relevant to the behavioral outcomes measure.

## Material and Method

### Animals

Male and female wild-type C57BL/6 mice were housed separately in groups of seven under standard conditions with unlimited access to food and water. Room temperature was maintained at 22 ± 3 °C with a 12/12-h light/dark cycle, and all behavioral experiments were performed during the light phase. We visually checked female mice for the estrous cycle, as we have shown that repeated collection of vaginal smears to determine estrous cycle causes stress in mice [16]. All animal experimental protocols were reviewed and approved by the Animal Experimentation Ethics Committee at Hacettepe University (2021/05–06).

### Acute Stress Procedure and Pharmacological Inhibitors

Male and female C57BL/6 mice were placed in rat cages inside a cylinder that restricted their movements and separated from the rat by a plexiglass wall, allowing visual, olfactory, and auditory exposure to the predator for 2 h [17]. NF-κB inhibitor (JSH23, 3 mg/kg, ADOOQ), a TLR4 inhibitor (TAK242, 3 mg/kg, ADOOQ), an HMGB1 inhibitor (glycyrrhizic acid, GA, 50 mg/kg, Sigma-Aldrich), or a vehicle (10% DMSO in saline) was administered intraperitoneally (ip) twice: first dose administered 1 day before, and the second dose administered an hour before the stress.

### Behavioral Experiments

#### Tail Suspension Test (TST)

Mice were suspended by their tails at a height of 80 cm above the floor for 6 min. The total duration of immobility during the final 4 min was measured. Increased immobility time indicates behavioral despair, a depression-like behavior. Test is scored manually by an investigator blind to the experimental groups.

#### Splash Test (ST)

After mice were sprayed on the back with a 10% sucrose solution, self-grooming behavior was recorded for 5 min. Reduced self-grooming indicates diminished self-care and anhedonia, thus increased depression-like behavior. Test is scored manually by an investigator blind to the experimental groups.

### Immunohistochemistry

After the behavioral tests, the mice were perfused with 4% PFA. Forty-micrometer-thick free float coronal brain sections were labeled with antibodies targeting NF-κB and HMGB1, iba-1, and cleaved caspase-1 (for NF-κB staining, antigen retrieval was applied in 1% citrate buffer at 80 °C for 15 min). Sections were permeabilized with PBST (Tween20) for 15 min. Sections were blocked in 10% normal goat serum (NGS) at room temperature for 1 h and then incubated with primary antibodies (anti-rabbit NF-κB (Cell Signaling) and anti-rabbit HMGB1 (Abcam), anti-rabbit iba-1 (Wako), anti-rabbit cleaved caspase-1 (p10 subunit) (Thermofisher) all at 1:200 dilution) in blocking solution overnight at 4 °C. After 1 h incubation with the secondary antibody (goat anti-rabbit, 1:200, in blocking solution, Jackson) at room temperature, sections were counterstained with a nuclear dye, Hoechst (33258), and mounted on slides. Images were taken using a confocal fluorescence microscope (Leica SP8) with ×25 objective or ×40 oil objective and were processed using FIJI [18]. Two-three coronal sections that are 320 µm apart, sampling the ACC and NAc, were selected for each animal. HMGB1 nuclear depletion, NF-κB nuclear translocation, and the cleaved caspase-1 ratio were computed by the following formulas, respectively:

- Number of HMGB1 *negative* nuclei/Total number of nuclei stained with Hoechst.
- Number of NF-κB *positive* nuclei/Total number of nuclei stained with Hoechst.
- Number of caspase 1 positive nuclei/Total number of nuclei stained with Hoechst.

For Sholl analysis, images were smoothed with a Gaussian blur (*σ* = 0.5); background was subtracted using a rolling ball radius of 50 pixels. Images were then binarized by auto-thresholding (Otsu algorithm). Noise reduction was performed (Remove Outliers (radius = 2 pixels, threshold = 50), Analyze Particles (minimum size = 10)). A median filter (radius = 5 pixels) was applied, and Sholl analysis was performed using the Sholl Analysis plugin [19]. Identical parameters were applied to all images. Images were analyzed by researchers blinded to groups.

### Statistical Analysis

For each brain region, we calculated the mean value from two–three sections per mouse and used it as the data point for statistical analysis. To evaluate sex and group effects on behavioral measures and immunohistochemical analysis, we used two-way ANOVA. Morphological analysis of microglia was performed by repeated measures of two-way ANOVA. Post hoc analysis was carried out by either Bonferroni test or Dunnett’s post hoc test.

## Results

### Behavioral Responses of Male and Female Mice Were Different Regardless of Acute Stress Effects

**Fig. 1:**
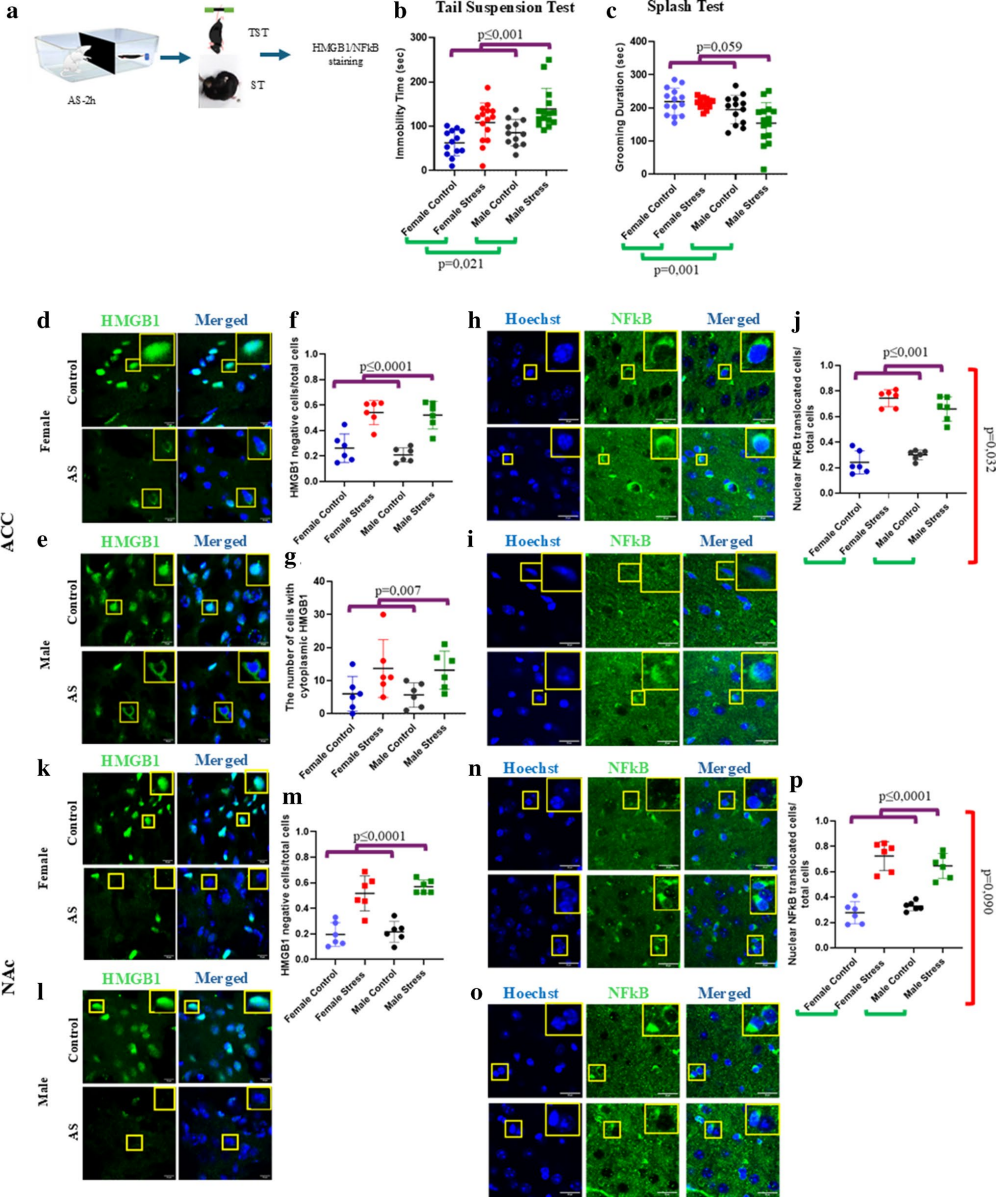
Sex-dependent effects of acute stress on depression-like behavior and inflammatory molecules. **a** Experimental design. Two h of restraint stress in a rat cage separated from the rat by a perforated divider. Tail suspension test (TST) and splash test (SP) were performed 15 min later. Brain sections were processed for HMGB1 and NF-κB immunostaining. **b** Acute stress effects on time spent immobile in tail suspension test in male and female mice. **c** Acute stress effects on grooming duration in splash test in male and female mice. **d**, **e** Representative immunofluorescent images of HMGB1 in the ACC from both sexes. Insets: The areas outlined by yellow rectangles are representative images of HMGB1. **f**, **g** Graphs showing stress-induced HMGB1 release and numbers of cytoplasmic HMGB1 number in the ACC in both female and male mice. **h**, **i** Representative immunofluorescent images of NF-κB in the ACC from both sexes. Insets: The areas outlined by yellow rectangles are representative images of NF-κB. **j** Graphs showing stress-induced nuclear translocation of NF-κB in the ACC in both female and male mice. **k**, **l** Representative immunofluorescent images of HMGB1 in the NAc from both sexes. Insets: The areas outlined by yellow rectangles are representative images of HMGB1. **m** Graphs showing stress-induced HMGB1 release in the NAc in both female and male mice. **n**, **o** Representative immunofluorescent images of NF-κB in the NAc from both sexes. Insets: The areas outlined by yellow rectangles are representative images of NF-κB. **p** Graphs showing stress-induced nuclear translocation of NF-κB in the NAc in both female and male mice. (For behavioral experiments *n* = 14/group and for IHC experiments *n* = 2–3 sections/mouse, 6 mice/group). Purple lines indicate stress effect; green lines indicate sex effect; red lines indicate sex × group interaction. Scale bars represent 20 µm

In the two-way ANOVA, we observed a sex effect on both TST immobility duration and ST self-grooming duration (*F*(1,52) = 5.653, *p* = 0.021 and *F*(1,52 = 12.217, *p* = 0.001). Male mice exhibited increased immobility in the TST and reduced grooming behavior in the ST when compared to female mice (Fig. 1b, c). In the TST, a significant effect of stress was observed (*F*(1,52) = 25.934, *p* < 0.0001). Both male and female mice showed increased immobility following acute stress exposure. In the ST, a non-significant trend towards a main effect of stress was observed (*F*(1,52) = 3.717, *p* = 0.059). Acute stress group tended to show decreased grooming duration in both sexes. No significant group × sex interaction was observed for either test (*F*(1,52) = 0.215, *p* = 0.645 for TST and *F*(1,52) = 2.266, *p* = 0.138 for ST) (Fig. 1b, c).

### HMGB1 and NF-κB Were Activated in ACC in Both Females and Males

In the two-way ANOVA, we observed a significant effect of stress on HMGB1 nuclear depletion in ACC (*F*(1,20) = 57.375, *p* < 0.001). Acute stressed mice had significantly less HMGB1-labeled nuclei than the controls, regardless of the sex. There was no significant sex effect on HMGB1 nuclear depletion (*F*(1,20) = 0.906, *p* = 0.353). No significant stress × sex interaction was detected (*F*(1,20) = 0.172, *p* = 0.683) (Fig. 1d–f). We also quantified the number of cells exhibiting cytoplasmic HMGB1 labeling. We found a significant main effect of stress (*F*(1,20) = 9.134, *p* = 0.007) but no significant main effect of either sex or stress × sex interaction (*F*(1,20) = 0.028, *p* = 0.870, and *F*(1,20) = 0.001, *p* = 0.974, respectively) (Fig. 1f).

There was a significant effect of stress on NF-κB nuclear translocation (*F*(1,20) = 193.794, *p* ≤ 0.0001). Acute stress increased NF-κB nuclear translocation (i.e., activation) regardless of the sex. There was no significant effect of sex on NF-κB nuclear translocation (*F*(1,20) = 0.171, *p* = 0.684). There was a significant main effect of stress × sex interaction, suggesting that NF-κB translocation to the nucleus in response to acute stress was different in males and females (*F*(1,20) = 5.297, *p* = 0.032) (Fig. 1h–j). In females, stress induced a threefold increase in NF-κB activation, whereas in males, it produced a 2.2-fold increase.

### HMGB1 and NF-κB Were Activated in NAc in Both Females and Males

There was a significant effect of stress on HMGB1 nuclear depletion in NAc, similar to that observed in ACC (*F*(1,20) = 74.593, *p* < 0.0001). HMGB1 was depleted from the nucleus by acute stress in both sexes. There was neither a main effect of sex (*F*(1,20) = 0.868, *p* = 0.363) nor a stress × sex interaction (*F*(1,20) = 0.179, *p* = 0.677 (Fig. 1k–m).

In NAc, we observed a significant effect of stress on NF-κB nuclear translocation (*F*(1,20) = 110.917, *p* < 0.001). Acute stress increased nuclear translocation of NF-κB in both sexes. There was no sex effect on NF-κ nuclear translocation (*F*(1,20) = 0.123, *p* = 0.730). There was a non-significant trend towards a stress × sex interaction on NF-κB nuclear translocation (*F*(1,20) = 3.175, *p* = 0.09). Acute stress tended to increase nuclear NF-κB translocation to a greater extent in females than in males (Fig. 1n–p).

### Inhibition of Inflammatory Signaling Reverses Acute Stress-Induced Behavioral Despair and Decreased Grooming in Males but not in Females

**Fig. 2:**
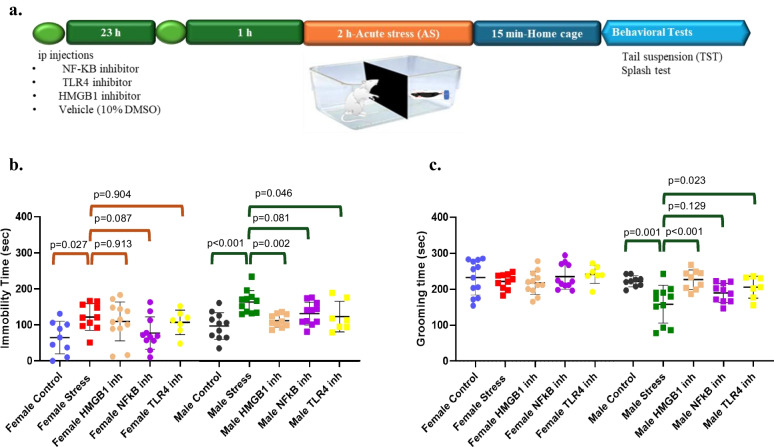
Effects of HMGB1, NF-κB, and TLR4 inhibitors on depression-like behaviors induced by acute stress. **a** Experimental design. HMGB1, NF-κB, and TLR4 inhibitors or vehicle were administered intraperitoneally 24 h and 1 h before acute stress, then the TST and ST were performed. **b** Graphs showing effects of HMGB1, NF-κB, and TLR4 inhibitors on immobility time in TST both female and male mice. A two-way ANOVA was used to determine sex × group interactions. ANOVA and post hoc Dunnett were used to compare group effects in each sex. **c** Graphs showing effects of HMGB1, NF-κB, and TLR4 inhibitors on grooming time in ST for both female and male mice. A two-way ANOVA was used to determine sex × group interactions. ANOVA and post hoc Dunnett were used to compare group effects in each sex. Data are presented as mean +/− SEM (*n* = 11–12/group)

To further explore whether there were sex-specific differences in acute stress-induced inflammatory signaling, pharmacological inhibitors of HMGB1, NF-κB, or TLR4 were administered prior to acute stress in both sexes (Fig. 2a). Although all inhibitors used are widely employed in the literature and are known to cross the blood-brain barrier [20–23], we confirmed their activity in the CNS by immunohistochemical analyses. In the HMGB1 inhibitor–treated mice, acute stress–induced increase in HMGB1 nuclear depletion was reduced approximately twofold. Similarly, in the NF κB inhibitor–treated mice, acute stress–induced increase NF κB nuclear translocation was reduced approximately 2.5-fold (Supplementary Fig. 1).

In two-way ANOVA, the main effects of sex (*F*(1,89) = 13.479, *p* < 0.001) and group (*F*(4,89) = 6.666, *p* < 0.001) on the immobility times in TST were significant. Females spent less time immobile than males regardless of the group. Acute stress increased time spent immobile compared to controls (*p* < 0.001). HMGB1 inhibitor and NF-κB inhibitor reversed acute stress–induced increase in immobility times (*p* = 0.022 and *p* = 0.005, respectively). On the other hand, TLR4 inhibitor was ineffective (*p* = 0.116). Main effect of sex-group interaction was found to be insignificant (*F*(4,101) = 1.463, *p* = 0.220). Univariate analysis separately in males and females revealed significant differences between treatment groups in males (*F*(4,45) = 6.490, *p* < 0.001) and in females (*F*(4,44) = 2.818, *p* = 0.036). Pairwise comparisons revealed a significant effect of HMGB1 inhibitor (*p* = 0.002) and TLR4 inhibitor (*p* = 0.046) but not NF-κB inhibitor (*p* = 0.08) in reversing acute stress–induced increase in immobility time in males. Pairwise comparisons in females revealed a significant difference between acute stress and non-stressed controls (*p* = 0.027), but none of the inhibitors was effective in reducing immobility times in females (*p* = 0.91 for HMGB1 inhibitor, *p* = 0.087 for NF-κB inhibitor, *p* = 0.90 for TLR4 inhibitor) (Fig. 2b).

In two-way ANOVA, the main effects of sex (*F*(1,88) = 18.074, *p* < 0.001) and group (*F*(4,88) = 3.857, *p* = 0.006) on the grooming duration in ST were significant. Female mice spent more time grooming than males. Acute stress decreased grooming time compared to controls (*p* = 0.001). HMGB1 inhibitor and TLR4 inhibitor reverse acute stress–induced decrease in grooming time (*p* = 0.009, *p* = 0.015, respectively). NF-κB inhibitor was ineffective (*p* = 0.07). Main effect of sex-group interaction was also found to be significant (*F*(4,88) = 3.559, *p* = 0.01). Univariate analysis separately in males and females revealed significant differences between treatment groups in males (*F*(4,41) = 6.714, *p* < 0.001), but not in females (*F*(4,47) = 0.752, *p* = 0.56) in accordance with our observation that acute stress did not affect grooming duration in females (Fig. 1b). Pairwise comparisons revealed a significant effect of HMGB1 inhibitor (*p* < 0.001) and TLR4 inhibitor (*p* = 0.023) but not NF-κB inhibitor (*p* = 0.13) in reversing acute stress–induced decrease in grooming times in males (Fig. 2c).

### Acute Stress Triggers Cleaved Caspase-1 (p10) Only in Male Mice

**Fig. 3:**
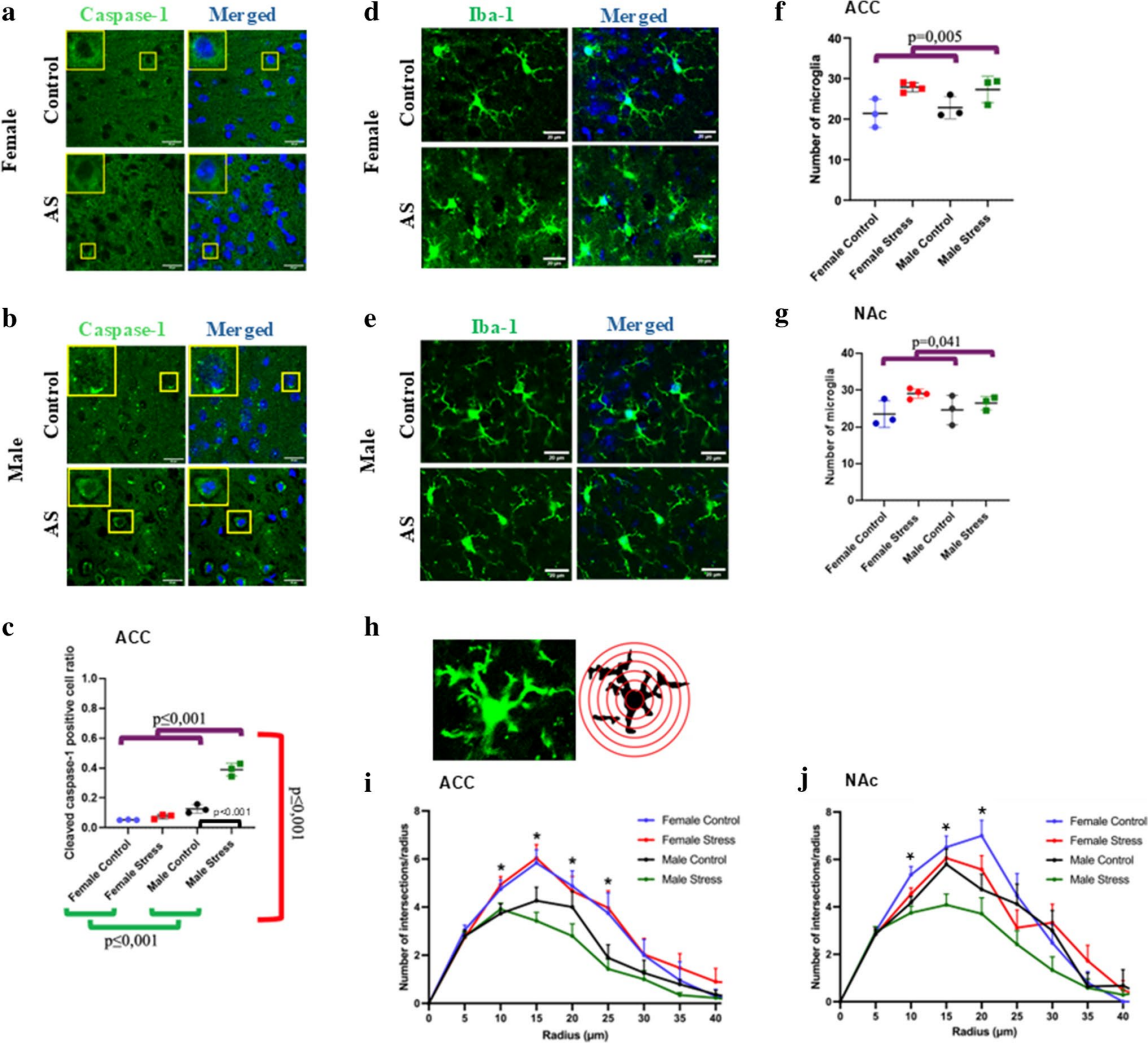
Sex-dependent effects of acute stress on cleaved caspase-1 and iba-1. **a**, **b** Representative images of cleaved caspase-1 staining in both sexes. Insets: The areas outlined by yellow rectangles are representative images of cleaved caspase-1. **c** Graphs showing effects of acute stress on active caspase-1 positive cell ratio in males and females. **d**, **e** Representative images of iba-1 staining in both sexes. **f**, **g** Graphs showing effects of acute stress on iba-1 numbers in ACC and NAc regions. Purple lines indicate stress effect, green lines indicate sex effect, and red line indicates sex × stress effect (*n* = 3 sections/mouse, 3–4 mice/group). **h**–**j** Sholl analysis was used to evaluate microglial branching in ACC and NAc. Scale bars represent 20 µm (*p < 0.05)

We then analyzed cleaved caspase-1 (p10) and microglial numbers, which serve as indicators of inflammasome activation and microglial activation, respectively, to investigate potential mechanisms underlying the sex-specific effects. A two-way ANOVA revealed a significant main effect of stress (*F*(1,10) = 84.09, *p* < 0.0001), sex (*F*(1,10) = 154.697, *p* < 0.001), and a significant stress × sex interaction (*F*(1,10) = 60.022, *p* < 0.0001). Cleaved caspase-1 (p10) levels were higher in males than in females (*p* < 0.001) and higher in AS group than the controls (*p* < 0.001). Stressed male mice exhibited higher levels of cleaved caspase-1 (p10) than control males in ACC (*p* < 0.001), consistent with activation of the inflammasome cascade, whereas stress had no effect on the levels of cleaved caspase-1 (p10) in females (*p* = 0.343) (Fig. 3a–c). We did not observe cleaved caspase-1 (p10) staining in NAc in any of the groups.

### Acute Stress Elicits Similar Microglial Recruitment Across Sexes but Induces Sex-Dependent Morphological Alterations

Two-way ANOVA revealed a significant main effect of stress in the ACC and NAc (*F*(1,10) = 13.196, *p* < 0.005, *F*(1,10) = 5.701, *p* = 0.041). Stressed mice, regardless of sex, showed increased numbers of microglia in both brain regions. There was neither a main effect of sex (*F*(1,10) = 0.075, *p* = 0.791 for ACC and *F*(1,10) = 0.238, *p* = 0.637 for NAc) nor a stress × sex interaction (*F*(1,10) = 0.450, *p* = 0.519 for ACC; *F*(1,10) = 1.452, *p* = 0.259 for NAc) (Fig. 3d–g).

In both ACC and NAc, microglial morphology showed a significant main effect of sex (*F*(7,721) = 3.024, *p* = 0.004, and *F*(7,826) = 2.039, *p* = 0.048, respectively, for ACC and NAc), with females exhibiting a more highly ramified morphology than males. There was no main effect of stress (*F*(7,721) = 0.443, *p* = 0.875, *F*(7,826) = 1.820, *p* = 0.08, respectively, for ACC and NAc), nor a significant stress × sex interaction (*F*(7,721) = 0.349, *p* = 0.931, *F*(7,826) = 0.957, *p* = 0.462, respectively for ACC and NAc). Post hoc comparisons showed that sex differences were localized at distances of 10, 15, 20 and 25 µm from the nucleus (*p* = 0.004, *p* < 0.001, *p* = 0.029, *p* = 0.001) in the ACC, and at distances of 10, 15, and 20 µm (*p* = 0.001, *p* = 0.012, *p* = 0.002) from the nucleus in the NAc.

## Discussion

**Fig. 4:**
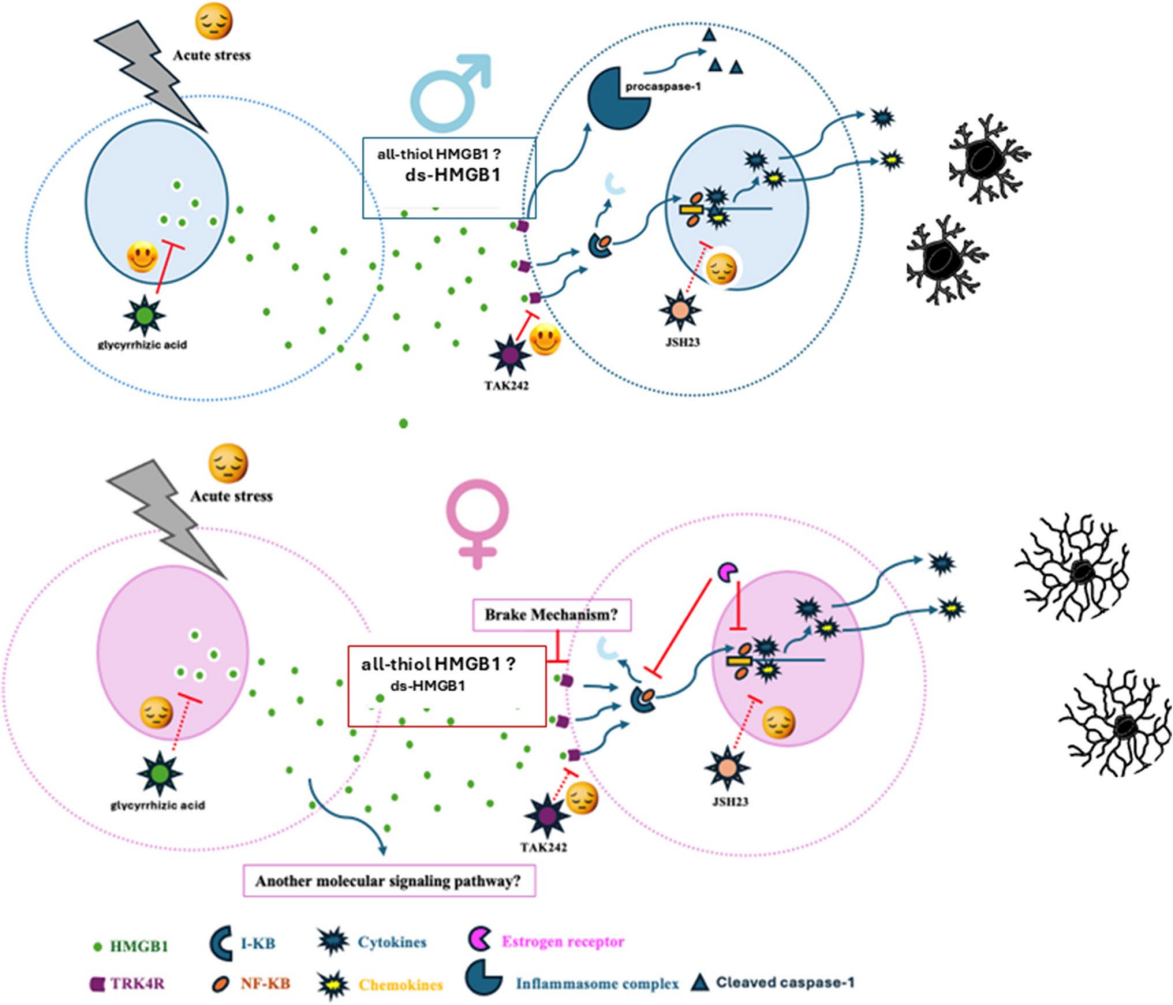
Summary of the findings: Acute stress triggers a comparable neuroinflammatory response in ACC and NAc in both sexes. Inhibition of HMGB1-TLR4-NF-κB pathway reverses acute stress induced behavioral despair and decreased grooming only in male mice indicating either the presence of potential brake mechanisms in females, which inhibit the proinflammatory response triggered by HMGB1-TLR4-NF-κB pathway or engagement of different secondary signal molecules by activation of HMGB1-TLR4-NF-κB pathway. Font size of ds-HMGB1 and all-thiol HMGB1 reflects their relative abundance

The present study demonstrates that acute stress activates the HMGB1–TLR4–NF-κB signaling pathway within the ACC and NAc in both male and female mice. Unlike our findings, evidence from the literature indicate a sexual dimorphism for HMGB1 release in response to physical stressors. Mouse and human pulmonary endothelial cells release HMGB1 in response to cellular stress in a male-specific manner, paralleling clinical findings that elevated circulating HMGB1 is observed in male but not female patients with pulmonary arterial hypertension [24–26]. Suggesting a similar sexual dimorphism in the CNS, addition of HMGB1 to male mice-derived primary microglial cell cultures induced higher levels of cytokine and chemokine expression when compared to female mice derived microglial cell culture [25]. Notably, inhibition of HMGB1 or its receptor TLR4 reversed acute stress–induced behavioral despair and reduced grooming behavior exclusively in males, with no significant effects observed in females, despite comparable activation of the HMGB1–TLR4–NF-κB pathway in both sexes (Fig. 4).

The effects of extracellular HMGB1 are shaped by the surrounding cytokine milieu and by cells expressing promiscuous receptors with broad ligand specificity [27]. Accumulating evidence indicates sex-dependent differences in both the cellular and extracellular milieu of male and female mice, including variations in sex hormones and their receptors, as well as in microglial number and morphology, which may, at least in part, contribute to the observed sex-specific differences [7, 28, 29]. One important difference relates to the effects of sex hormones, particularly estrogen and its receptors. HMGB1 has been reported to enhance the binding affinity of estrogen receptors (ERs) to estrogen response elements, as well as the binding of NF-κB to its target DNA sequences [30–32]. In addition, there is well-documented crosstalk between ER and NF-κB signaling pathways. ERs have been shown to inhibit NF-κB activity through multiple mechanisms, including modulation of IKK activity or IκB degradation in the cytoplasm, as well as interference with NF-κB–mediated transcription in the nucleus [33]. Given that psychological stress has been associated with reduced estrogen levels in women [22], it is possible that this may lead to alterations in NF-κB signaling and downstream cytokine expression, including IL-1, IL-6, and TNF-α [22]. Together, these observations raise the possibility that estrogen-related mechanisms may modulate the effects of HMGB1–TLR4–NF-κB pathway inhibition in females, although this remains to be directly tested.

Notably, the biological activity of HMGB1 is highly dependent on its redox state: disulfide HMGB1 (ds-HMGB1) preferentially signals through TLR4, whereas all-thiol HMGB1 interacts with CXCL12 and its receptor CXCR4 [34] [35, 36] [37]. Thus, the proinfl ammatory activity of HMGB1 depends on the disulfi de linkage between cysteine residues Cys23 and Cys45 within the Box A domain [35], and it is possible that HMGB1 in females may be found in different redox states than in males Estrogen’s role in restoring redox balance through its antioxidant properties supports this assumption [38]. Through CXCL12–CXCR4 signaling, HMGB1 can function as a chemoattractant. To investigate whether acute stress–induced HMGB1 engages different receptor pathways in males and females, we analyzed microglial number and morphology, as microglia are recruited to sites of injury by chemoattractant signals. Although stress increased microglial numbers in both sexes, microglial morphology showed a significant main effect of sex but no main effect of stress or stress × sex interaction, with females exhibiting a more highly ramified morphology than males, consistent with previous reports [28, 29]. These findings suggest that microglial recruitment in response to HMGB1 is unlikely to account for the observed sex differences. However, the distinct morphological profiles may reflect functional differences in the recruited microglia. A recent study provide evidence for the involvement of microglial MyD88 signaling in the anxiety-inducing effects of ds-HMGB1 specifically in females [39]. In contrast to our findings, that study reported increased microglial reactivity in female mice following repeated infusions of ds-HMGB1 into the medial prefrontal cortex over 5 days. Moreover, conditional deletion of microglial MyD88 attenuated the anxiogenic effects of repeated ds-HMGB1 infusions in females, but not in males. The discrepancies between our findings and those of Rawls et al. (2025) may be attributable to methodological differences, including acute versus chronic elevations in HMGB1, local versus systemic manipulations, the specific mPFC subregions targeted, and the possibility of supraphysiological HMGB1 concentrations in the mPFC [39].

Another potential explanation for the sex-specific effects of the inhibitors is that, in females, HMGB1 may preferentially exert its effects within the cytoplasm rather than being released extracellularly in response to acute stress. Cytoplasmic HMGB1 has unique functions; specifically, it induces autophagy and mitochondrial clearance by autophagy in response to cellular stress [40–42]. However, we found no sex-specific differences in cytoplasmic HMGB1, suggesting that the location of action of HMGB1 is unlikely to account for the observed sex-dependent effects. Further studies are needed to delineate the downstream targets of HMGB1 and to identify mechanisms that may counteract its depressogenic effects in females.

The only inflammatory molecule investigated in this study that was altered by acute stress in a sex-specific manner is cleaved caspase-1. At basal conditions, levels of cleaved caspase-1 were low in both sexes. Following acute stress exposure, cleaved caspase-1 was increased ~ threefold in male mice, but not in females. Caspase-1 is a central effector component of the inflammasome complex, where it is recruited and activated through proximity-induced autocleavage upon inflammasome assembly. Caspase 1 not only induces HMGB1 release from immune cells but also cleaves HMGB1 with the end product having proinflammatory effects [43, 44] [45]. This sex-specific effect of acute stress on cleaved caspase-1 further supports the notion that the proinflammatory actions of HMGB1, determined by its redox state, may predominate in males.

Regarding the limitations of the study, changes in neuroinflammatory signaling were assessed only in ACC and NAc. These regions were selected based on their well-established associations with the behavioral paradigms used to assess depression-like behavior [14, 15, 46]. Nonetheless, sex-specific behavioral effects of acute stress and differential response to pharmacological inhibitors observed in this study may result from changes in neuroinflammatory alterations in other brain regions and should be addressed in future studies. In addition, this study focused exclusively on acute stress; thus, future studies are needed to determine whether there are similar sex-specific differences in response to chronic stress. The number of mice used for pharmacological experiments were small, although comparable to similar studies [39, 47]. All female mice were in their diestrus phase during the experiments, which limits the generalizability of the findings to other phases of the menstrual cycle. Finally, as we did not inject pharmacological inhibitors to non-stressed groups, we cannot rule out the possibility of a sex-dependent effect of inhibitors on baseline behavior. There is evidence for lack of effect of glyzcyrrhizic acid on TST results in control male mice [48], but data in female animals is largely lacking.

In conclusion, we showed that although acute stress produced depressogenic effects and increased HMGB1 release and NF-κB activation in both sexes, the efficacy of pharmacological inhibitors targeting different components of neuroinflammatory signaling depended on sex. Together, these findings raise the possibility that HMGB1 and NF-κB may be linked to distinct pathways in males and females, or that their association with depression-like behavior in females may be modulated by counteracting mechanisms. Our results further support the recent emphasis on the inclusion of both sexes in research, which is essential for the development of sex-specific therapeutic interventions for stress-related mental disorders.

## Supplementary Information

Below is the link to the electronic supplementary material.

ESM 1 Supplementary Fig. 1. Confirmation of central effects of NF-κB and HMGB1 inhibitors. (a) Representative immunofluorescent images of NF-κB labeling. (b) NF-κB inhibitor reversed the acute stress-induced nuclear translocation of NF-κB, confirming its central effect. (c) Representative immunofluorescent images of HMGB1 labeling. (d) HMGB1inhibitor reversed the acute stress-induced HMGB1 depletion from the nucleus, confirming its central effect (*n *= 3 sections/mouse, 3 mice/group). Scale bars represent 20 µm (*p ≤ 0.05) (PNG 1.85 MB)

ESM 1 High Resolution Image (TIF 431 KB)

## Data Availability

Data are available from the authors upon reasonable request.
