# Supplementary figures and images for: Sex-Specific Reversal of Stress-Induced Depressogenic Behaviors by Inhibition of HMGB1, TLR4, and NF-κB Signaling
Source: Mol Neurobiol. 2026 May 6;63(1):611. doi: 10.1007/s12035-026-05908-7 (PMC13144238; doi:10.1007/s12035-026-05908-7)

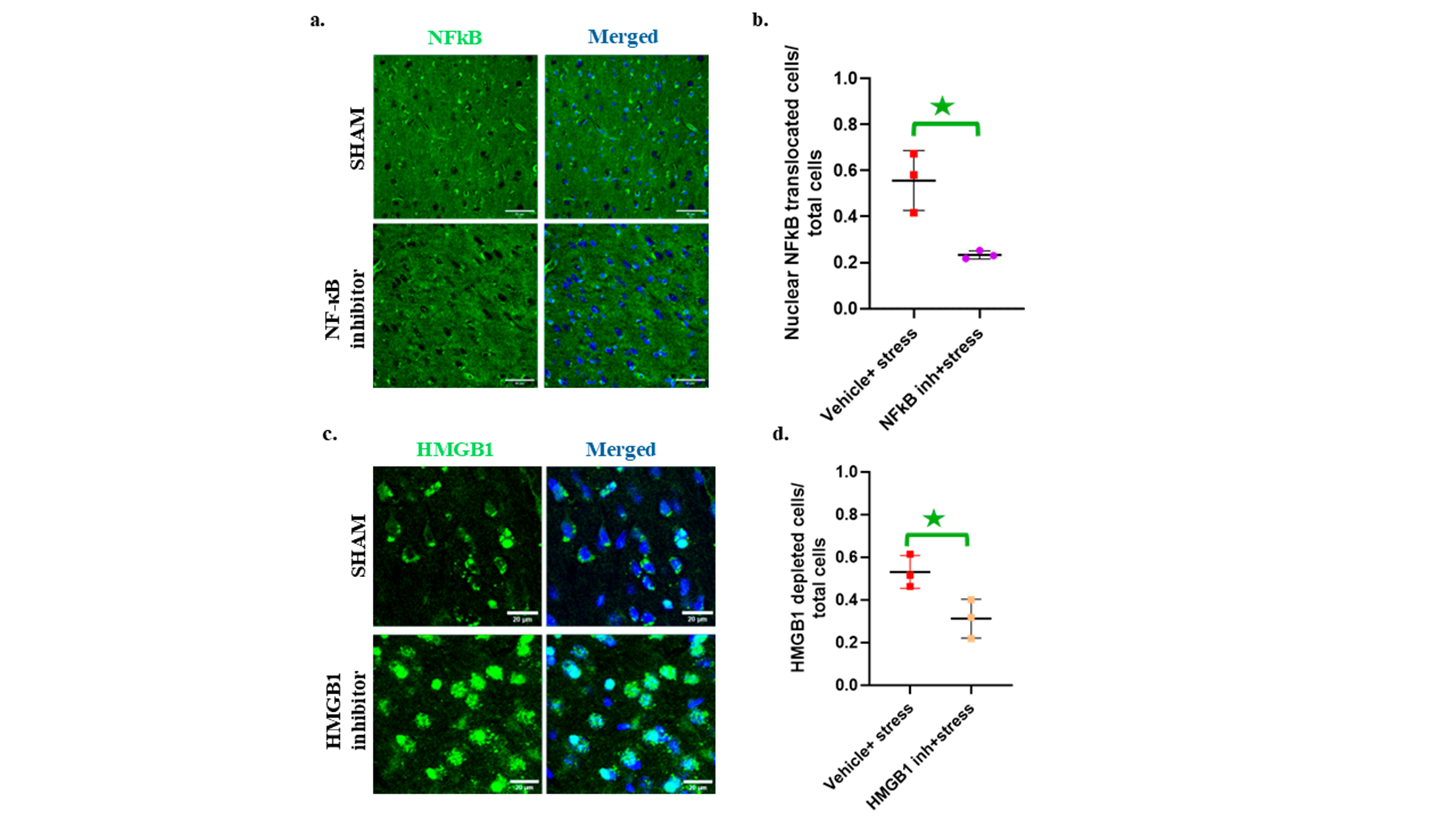

Supplement: Supplementary file 1 — Supplementary Fig. 1. Confirmation of central effects of NF-κB and HMGB1 inhibitors. (a) Representative immunofluorescent images of NF-κB labeling. (b) NF-κB inhibitor reversed the acute stress-induced nuclear translocation of NF-κB, confirming its central effect. (c) Representative immunofluorescent images of HMGB1 labeling. (d) HMGB1inhibitor reversed the acute stress-induced HMGB1 depletion from the nucleus, confirming its central effect (n = 3 sections/mouse, 3 mice/group). Scale bars represent 20 µm (*p ≤ 0.05) (PNG 1.85 MB) [file 12035_2026_5908_Fig5_ESM.png]

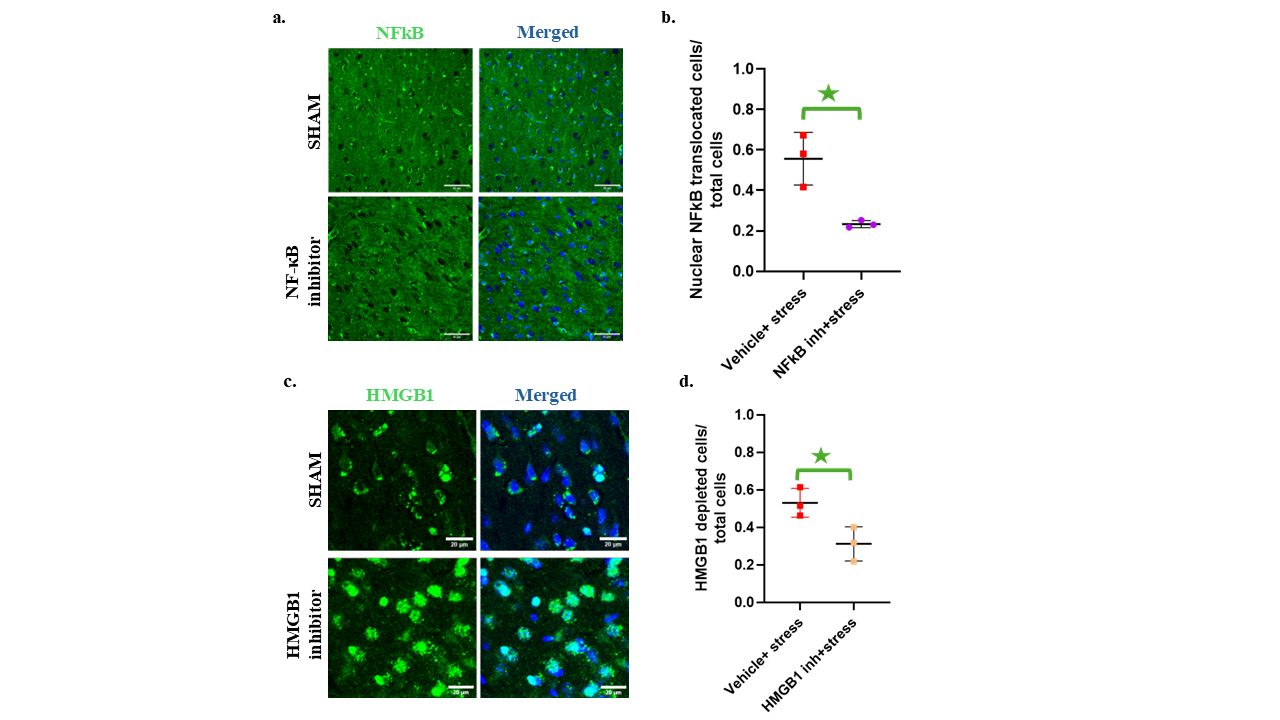

Supplement: Supplementary file 2 — High Resolution Image (TIF 431 KB) [file 12035_2026_5908_MOESM1_ESM.tif]
